# Epidemiological characterization of dermatophytes at a tertiary care hospital in Eastern Uttar Pradesh, India

**DOI:** 10.18502/cmm.5.1.530

**Published:** 2019-03

**Authors:** Vandana Upadhyay, Ankur Kumar, Amresh K. Singh, Jayesh Pandey

**Affiliations:** 1 Department of Microbiology, Baba Raghav Das Medical College, Gorakhpur, Uttar Pradesh, India

**Keywords:** Dermatophytes, Trichophyton verrucosum, Lactophenol cotton blue (LCB), Sabouraud’s dextrose agar medium, Tinea corporis

## Abstract

**Background and Purpose:**

Superficial mycosis is more prevalent in tropical and subtropical countries, such as India. Regarding this, the present study was conducted to determine the epidemiology of superficial mycosis and identify the most common dermatophytic species in this region.

**Materials and Methods:**

For the purpose of the study, a total of 220 skin scraping, nail, and hair root specimens were collected. Direct microscopic examination was performed using potassium hydroxide mount. Additionally, the samples were inoculated onto Sabouraud dextrose agar (SDA) and dermatophyte test medium (DTM). The fungal colony of each isolates was stained with lactophenol cotton blue mount, and observed under microscope for species identification.

**Results:**

Out of 220 isolates, 172 samples, obtained from 108 males 64 females, were positive for skin fungal infections by either KOH mount or culture. Furthermore, 113 isolates were identified as dermatophytes, while 59 samples were found to be non-dermatophytes. Among the dermatophytes isolated from different clinical samples,* Trichophyton verrucosum* (42/113, 38%) was the most common species, and *Tinea corporis* was the most common infection (36.2%).

**Conclusion:**

As the findings indicated, dermatophytes had an isolation rate of 78%, which is higher than normal. This can be due to the fact that the majority of the patients were from a rural background (71.7%) with a low socioeconomic status and poor personal hygiene who were exposed to climatic changes.

## Introduction

Superficial mycoses refer to the diseases of the skin, nail, and hair caused by fungal agents. Over the past decades, the prevalence of these infections has been on a rising trend; accordingly, they have affected 20-25% of the world’s population. These diseases are more common in the tropical countries due to humidity, elevated temperature, and sweating. The major examples of superficial mycoses include dermatophytosis, pityriasis versicolor, and candidiasis [1]. Superficial mycosis is more prevalent in tropical and subtropical countries, such as IndiaThe clinical lesions caused by the fungi are highly variable and closely resemble other skin diseases. Therefore, it is necessary to make a confirmed laboratory diagnosis of superficial skin infection [2]. Dermatophytes are hyaline septate moulds with more than 100 species. Nearly 40% of dermatophytes account for the incidence of other medical illnesses. According to Emmon’s morphological classification, dermatophytes are classified into three major genera, including *Epidermophyton*, *Microsporum, *and* Trichophyton* on the basis of their conidial morphology [3].

The severity of dermatophytic infections depends on a variety of factors, such as host reactions to the metabolic products of the fungi, virulence of pathogenic species or particular strain, anatomical site of the infection, and local environmental risk factors [4]. Dermatophytes are mainly aerobic fungi producing enzyme-like proteases that digest keratin and permit colonization, invasion, and infection of the skin, hair shaft, and nails.

Dermatophytosis is mainly confined to the non-living superficial cornified layers because its fungal agents are not able to penetrate into the deeper tissue or organ of a healthy host. However, this infection also depends on the fungi, immune status of the host, and site of infection [5]. The dermatophytic infection spreads easily by direct contact with the infected humans and animals or through fomites. Although the infection is non-invasive and curable, its widespread nature and therapeutic costs are major public health problems, imposing a high economic burden on society, especially in the developing tropical countries like India [6].

The enhancement of the degree of immune-suppression and number of immunosuppressed patients would confront the mankind with a definitive challenge in the coming years. There are an increasing number of epidemiological studies on dermatophytosis in India; accordingly, this issue has been given attention in recent years. The prevalence of the fungal infection of the skin is on a growing trend in India due to its climatic conditions, such as its temperature and humidity setting the ground for the spread of this infection. 

Therefore, the prevalence of superficial mycosis is near about 20 -25% worldwide and dermatophytes being the leading cause of superficial fungal infection [7]. The current upsurge of complicated dermatophytosis in India particularly the tropics due to the fact that dermatophytosis is a predominantly tropical dermatosis with this background in mind, the aim of present study was to understand the epidemiology of dermatophytic infection and to identify the species of fungi that are prevalent in this region.

## Materials and Methods

This prospective study was conducted in the Department of Microbiology and Dermatology of Baba Raghav Das Medical College and Nehru Hospital, Gorakhpur, Uttar Pradesh, India, from June 2017 to May 2018. Skin scraping, nail, hair root, and superficial pus samples were collected from the patients attending hospital with the suspicion of dermatophytic infection.

The clinical and demographic data recorded during sample collection included the mean size of the indurated lesions, clinical symptoms, kind of administered therapy, age, gender, and duration of illness. 

The study was approved by the Ethics Committee of Institute (gazette No. BRDMC/1339/2017) as per Ministry of Health, Government of India. The specimens were subjected to direct microscopic examination for the presence of fungal agents using 10% potassium hydroxide (KOH) in HiMedia Laboratory, Mumbai, India [7]. 

The samples were also cultured on Sabouraud dextrose agar (SDA) medium (HiMedia laboratory, Mumbai, India), containing chloramphenicol (0.004%) and cycloheximide (0.05%) to prevent bacterial contamination, and also dermatophyte test medium (DTM; HiMedia Laboratory, Mumbai, India). The inoculated SDA tubes were incubated at 25°C and 37°C for 4-5 weeks, and then examined for fungal growth. The DTM was incubated at 25°C for the appropriate growth of dermatophytes. This medium is used to isolate the dermatophytes that could not grow on SDA media. This medium also facilitates the isolation of dermatophytes as pure growth from the bacterial contaminants found in the cutaneous lesions [8]. 

**Figure 1:**
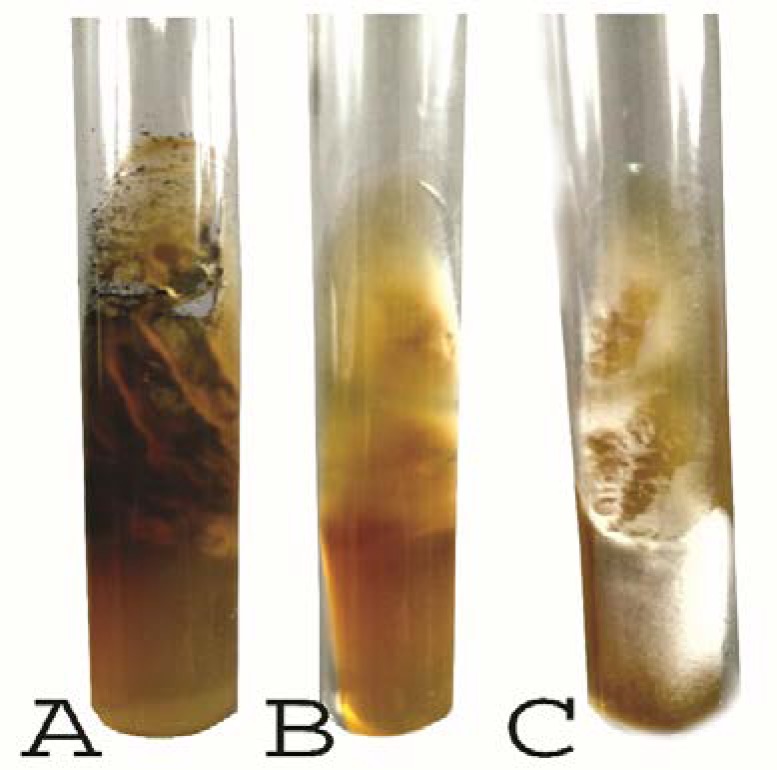
Growth on Sabouraud dextrose agar medium; (A) *Trichophyton tonsurans* (obverse side), (B) *Trichophyton tonsurans* (reverse side), and (C) *Trichophyton mentagrophytes*

**Figure 2:**
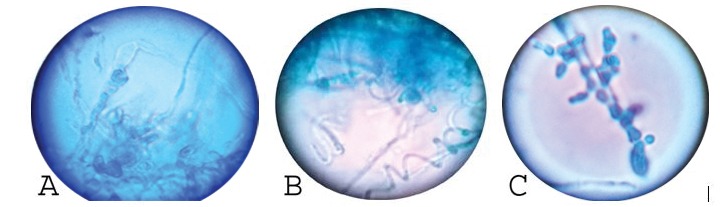
Lactophenol cotton blue mount showing the morphology of (A) *Trichophyton verrucosum,* (B) *Trichophyton mentagrophytes*, and (C) *Trichophyton tonsurans*

**Figure 3:**
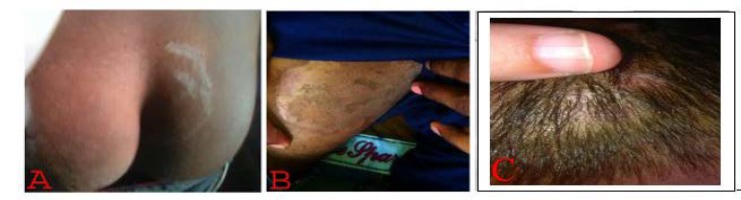
Clinical picture of dermatophytic infection (A) *Tinea* *corporis* (B) *Tinea cruris* (C) *Tinea capitis*

The fungal growth on the culture medium was identified on the basis of colony characteristics and pigmentation (both on the obverse and reverse sides). The final confirmation was performed using lactophenol cotton blue for staining (HiMedia laboratory, Mumbai, India), hair perforation test, and biochemical tests. The *Trichophyton* *species* were also subjected to urease test on Christensen’s medium (HiMedia laboratory, Mumbai, India) for the differential identification of *T. mentagrophytes* (showing a positive result) and *T. rubrum *(having a negative result). The identification of dermatophytes was accomplished using fungal morphology, including hyphal organization (i.e., septate or aseptate), as well as the presence and arrangement of microconidia and macroconidia [10] (figures 1, 2, and 3). The data were analysed using Chi-square test, and the examination of the hypothesis was performed in Microsoft excel. 

## Results and Discussion

Out of the 220 suspected cases of superficial mycosis examined for the different types of dermatophytic infections, of clinical samples 168 from skin, 35 from hair, and 17 from nails were collected in a hospital Among a total of 220 clinical samples obtained; 168 from skin, 35 from hair and 17 were from nails. 139 (82.73%), 25 (71.4%) from hairs and 08 (47%) from nails were positive by any method either by microscopy or culture shown in table 1. Maximum fungal isolates were positive in skin samples by KOH microscopy (135) culture positive (126) which is similar to other studies [1, 8]. Out of 220 samples 166 were KOH positive by direct microscopy and 149 were culture positive. (Table 1,3) Furthermore, 166 and 149 samples were KOH- (in direct microscopy) and culture-positive for dermatophytes, respectively. Our results revealed a high incidence of dermatophytic infection among all positive cases for superficial infection. Svejgaard et al. also obtained a similar finding in a study performed in Singapore where more than 2,500 cases of superficial fungal infections are annually reported [11].

**Table 1:** Distribution of clinical samples and result of microbiological investigation

**Site**	**No. of samples**	**KOH-positive samples**	**Culture -positive samples**
Skin scraping	168	135	126
Hair follicle	35	24	19
Nail	17	07	4
Total	220	166	149
Percentage	%	75.45	67.72

**Table 2:** Distribution of microbiological results as per different age groups

**Age group**	**Total**	**Sex**		**KOH positive**	**Culture positive**
		**Male**	**Female**		
0 – 5 years	3	2	1	2	1
6 – 20 years	52	35	17	54	49
21 – 50 years	104	63	41	99	87
>50 years	13	8	05	11	12
Total (n=113+59=172)	172	108	64	166	149

**Table 3:** Comparison and distribution of findings of potassium hydroxide and culture among positive samples

**KOH and Culture Observation among positive samples**		
**Findings**	**Number of patients**	**percentage**
KOH Positive, Culture Positive	146	66.36
KOH Positive, Culture -Negative	15	6.8
KOH Negative, Culture Positive	41	18
KOH Negative, Culture -Negative	06	2.7
Total KOH/Culture positive sample	172	78.18

However, the prevalence rates of positive cases for dermatophytes were lower in a couple of studies conducted by Svejgaard et al. and Doddamani et al., compared to the rate obtained in the present study [11, 12] (Table 1). Out of 220 samples, 113 cases were identified as dermatophytes, while 59 specimens were non-dermatophytes.

***Demographic characteristics of participants***

The samples were obtained from 125 males and 95 females. None of the subjects had any systemic diseases or comorbid condition. Out of 220 isolates, 172 (108 males and 64 females) cases were found positive by either KOH mount or culture. In our study, males (63%) were more prone to superficial fungal infections than female (37%). Accordingly, these infections had a male to female ratio of 1.7:1, which is similar to the ratio reported in other studies conducted elsewhere in India, such as those performed by Chaudhary et al., Doddamani et al., and Singh et al. [8, 12, 13]. 

In the current study, the most and least common affected age groups were 21-50 (n=104, 47%) and 0-5 (n=3, 1%) years, respectively, which is in line with the results obtained by Chaudhary et al., Bhavsar et al., and Grover et al. performing studies in Bihar, Gujarat, and north east states of India [8, 9, 14]. A higher frequency of this infection in adult age group indicates that the involvement in outdoor activities, which leads to excessive sweating in a warm humid condition, make a favourable environment for fungal infections.

As the results indicated, 78.18% of the samples were positive for superficial mycosis either through direct microscopic examination or culture. Furthermore, a total of 146 (66%) cases were positive for this infection by both KOH and culture techniques. Furthermore, only 6 (2.7%) samples were found negative by both KOH and culture methods, which is similar to the result reported by Chaudhary et al. in Bihar [8] (Table 3). 

***Distribution of dermatophytes***

**Figure 4:**
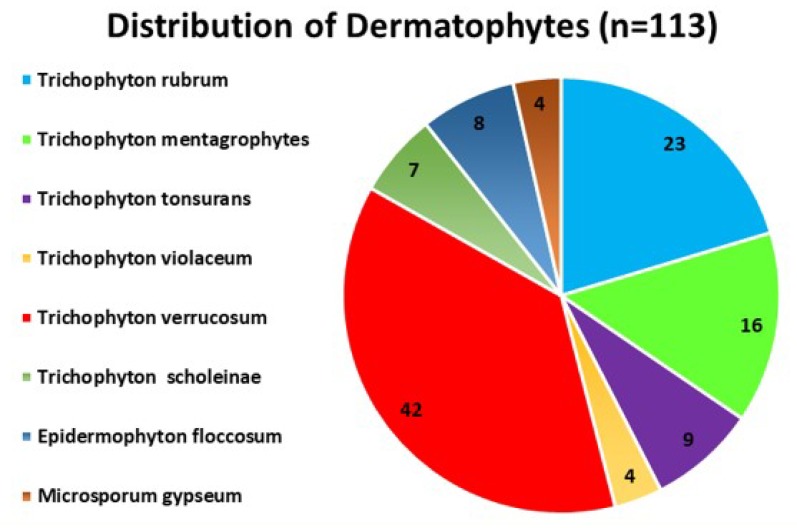
Distribution of Dermatophytes (n=113) among positive cases

**Figure 5:**
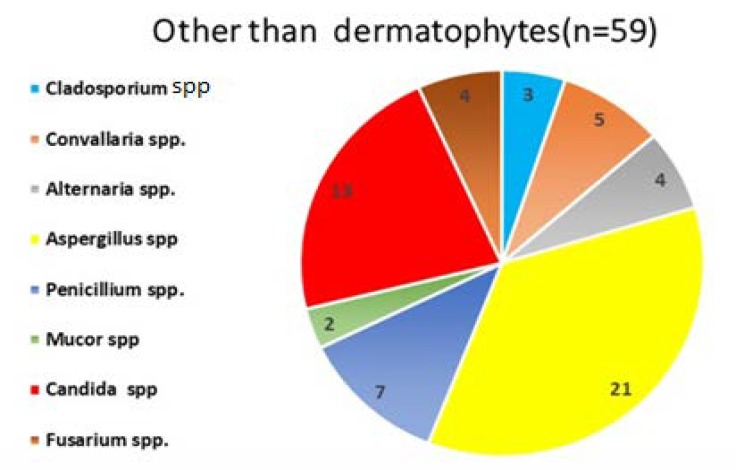
Distribution of non-dermatophytes (n=59) among positive cases

A total of 139 (42.5%) culture-positive isolates were subjected to further investigation. Out of these culture-positive isolates, 113 cases were identified as dermatophytes. The most common dermatophytes were *T. verrucosum* (n=42, 38%), followed by *T. rubrum* (n=23, 20%), *T. mentagrophytes* (n=16, 14%), *T. tonsurans* (n=9, 8%), *Epidermophyton floccosum* (n=8, 7%),* T. schoenleinii* (n=7, 6%), *T. violaceum* (n=4, 3%), and *Microsporum gypseum* (n=4, 3%). Regarding the non-dermatophytes, *Aspergillus species* (n=29) were the most common fungi, followed by *Candida *species (n=13), *Alternaria *species (n=2), and *Curvularia *species (figures 4 and 5).

As stated, *T. verrucosum *(38%) was the most common isolated dermatophyte*. *This is probably due to the proximity of the affected individuals with cattle, which have ringworm infection. Our observations are quite different from those in other studies performed by Doddamani et al. (in Madhya Pradesh, India), Singh et al. (in Gujarat, India), and Aggarwal et al. (in Punjab state, India), reporting a low frequency of *T. verrucosum *[12, 13, 15]. However, Patel et al. reported the isolation of *T. verrucosum* from human ringworm infection in other parts of India [16]. According to Menon et al., about 80% of human ringworm infections in rural areas are of animal origin [17].

***Distribution of clinical types***

*Tinea corporis* was the most common infection (n=421, 36.2%), followed by *T. cruris* (n=17, 15%), *T. capitis* (n=13, 11.5%), *T. unguium* (n=11, 9.7%), *T. barbae* (n=6, 5.3%), *T. magnum* (n=4, 3.5%), *T. pedis* (n=4, 3.5%), and *T. faciae* (n=3, 2.6%). Additionally, 14 cases were found to be mixed fungal infections (Table 4 and figures 3, 4, and 5). 

**Table 4:** Correlation of different clinical types of dermatophytic infection and etiological characterization

**Fungal pathogen**	***T. *** ***rubrum***	***T. *** ***tonsurans***	***T. verrucosum***	***T. violecum***	***T. schoeleini***	***T. mentagrophytes***	***E. floccosum***	***M. gypseum***	**Total**
*T. corporis*	5	04	17	0	03	07	02	03	41
*T. cruris*	5	0	04	0	0	03	04	01	17
*T. capitis*	3	02	0	03	01	04	0	0	13
*T. unguinum*	2	2	04	0	02	01	0	0	11
*T. barbae*	2	0	03	0	0	01	0	0	06
*T. mannum*	3	0	0	01	0	0	0	0	04
*T.pedis*	2	0	01	0	0	0	01	0	04
*T. faciae*	1	0	02	0	0	0	0	0	03
Mixed	0	1	11	0	1	0	01	0	14
Total	23	09	42	04	07	16	08	04	113
%	20	8	38	3	6	14	7	3	100

In the current study, *T. corporis* (36.2%) and *T. faciae* (2.6%) were identified as the most and least common clinical forms of dermatophytic infections. The prevalence of *T. corporis* in other studies conducted in different parts of India, including Karnataka, Bihar and Gujarat, have been reported as 44.3%, 27%, and 18.3%, respectively [5, 8, 18]. Similar study was conducted in Iran and Lebanon, as they have reported *T. corporis* was the most common clinical manifestation by Rassai et al. and Araj et al. [19, 20].

As the findings of the present study indicated, *T. verrucosum* and *T. mentagrophytes* were highly frequent in cases with *T. corporis*, *T. cruris*, and *T. unguium*. The presence and adaptability of these species to environmental conditions and their close contact with human promote the fungal invasion whenever they dwell in human [21, 22]. Therefore, in the present study, the ringworm infections caused by *T. verrucosum *and* T. mentagrophytes* were identified as the most common infections in the investigated geographical region. This can due to the fact that most of the patients investigated in the present study were from rural areas and had a close contact with cattle for dairy and farming, which are the major occupations in the villages. Moreover, the other risk factors, including lower socioeconomic status and lack of awareness about the zoophilic infections can contribute to the high prevalence of these infections. 

In this regard, ringworm infections can be acquired through direct contact with infected animals or indirect contact with contaminated fomites. These zoophilic dermatophytes have been also isolated from the skin lesions of labour workers from different parts of the world. This underscores the importance of these dermatophytes in the development of human ringworm infections.

Indian subcontinent has a very diverse climatic condition. Topographical climate is conducive to the acquisitions of mycotic infections. The findings of the present study revealed that different species of *Trichophyton* were responsible for the incidence of the majority of the dermatophytic infections. This finding is in line with those reported by other researchers from different parts of the world [5, 8, 9].

## Conclusion

Dermatophytes are the most common agents accounting for cutaneous fungal infections mostly occurring in the tropical and subtropical areas, such as India. Prevalence of dermatophytic infections depends on environmental factors, personal hygiene, and individual susceptibility. In the current study, dermatophytes had an isolation rate of 78%, which is quite higher than the rates reported in other studies. This can be due to the fact that the majority of the investigated patients in Gorakhpur, India, and its surrounding regions were from rural areas where poor living standards and unhygienic condition predispose the inhabitants to a high risk of cutaneous fungal infections.
